# Supplementary figures and images for: Molecular basis of P[II] major human rotavirus VP8* domain recognition of histo-blood group antigens
Source: PLoS Pathog. 2020 Mar 24;16(3):e1008386. doi: 10.1371/journal.ppat.1008386 (PMC7122821; doi:10.1371/journal.ppat.1008386)

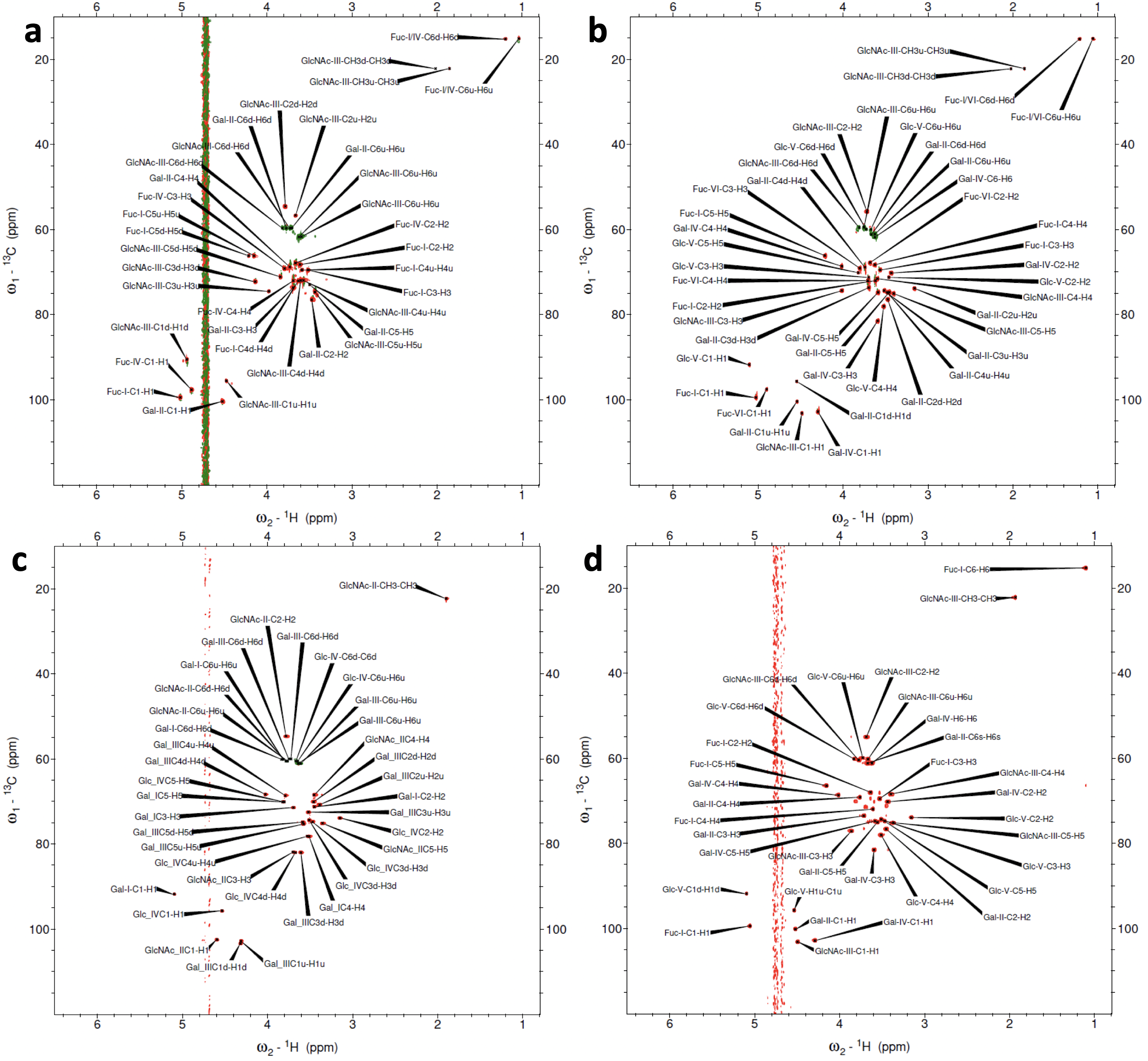

Supplement: S1 Fig — Two-dimensional heteronuclear single quantum coherence spectroscopy of (a) Leb tetra-saccharide. (b) LNDFH I. (c) LNT. (d) LNFP I. Individual resonance assignments are indicated by labels. Red colors indicate positive signals, green colors indicate negative signals. (TIF) [file ppat.1008386.s001.tif]

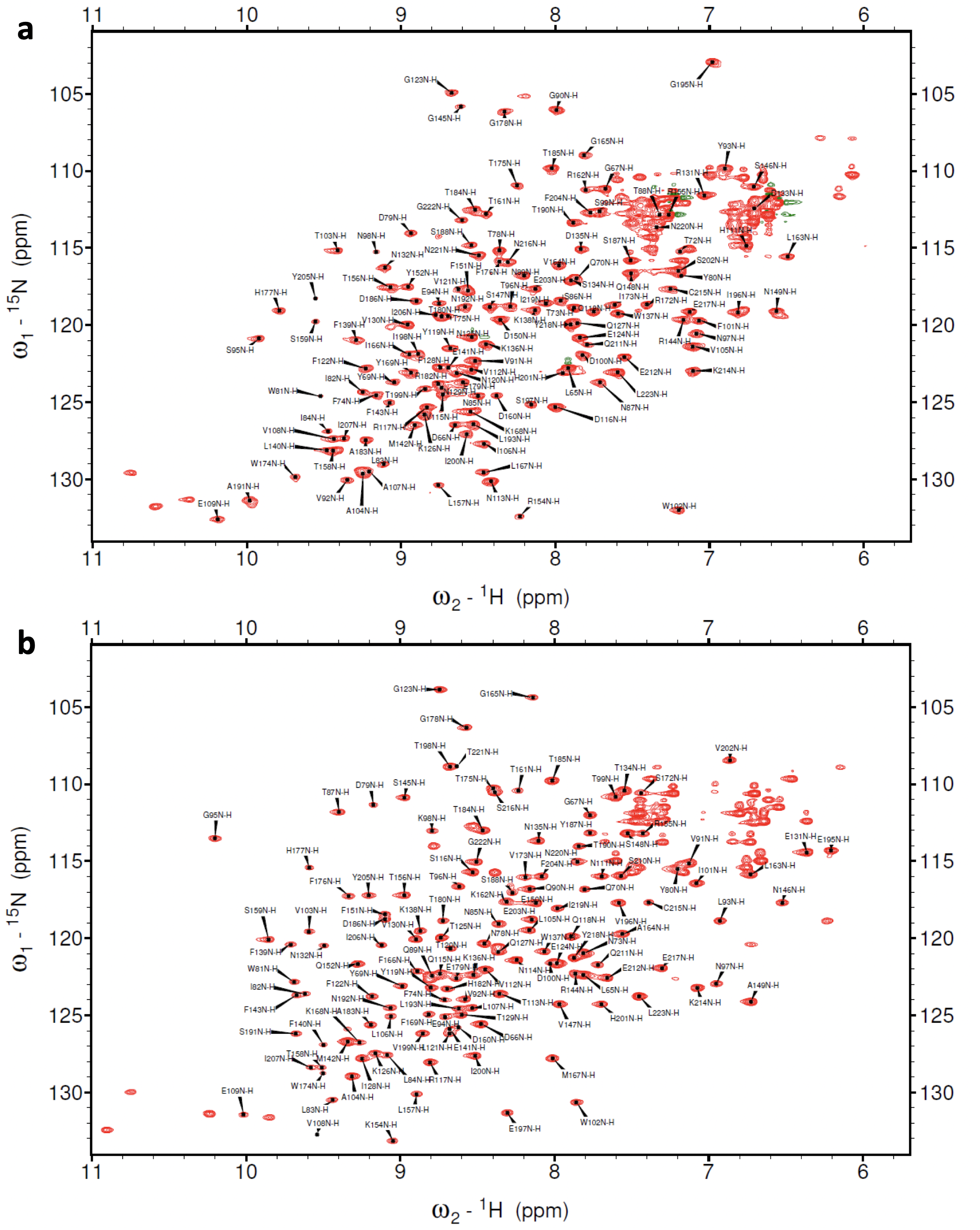

Supplement: S2 Fig — The 1H-15N HSQC spectrum of (a) P[8] VP8* and (b) P[6] VP8*. Individual resonance assignments are indicated by labels. (TIF) [file ppat.1008386.s002.tif]

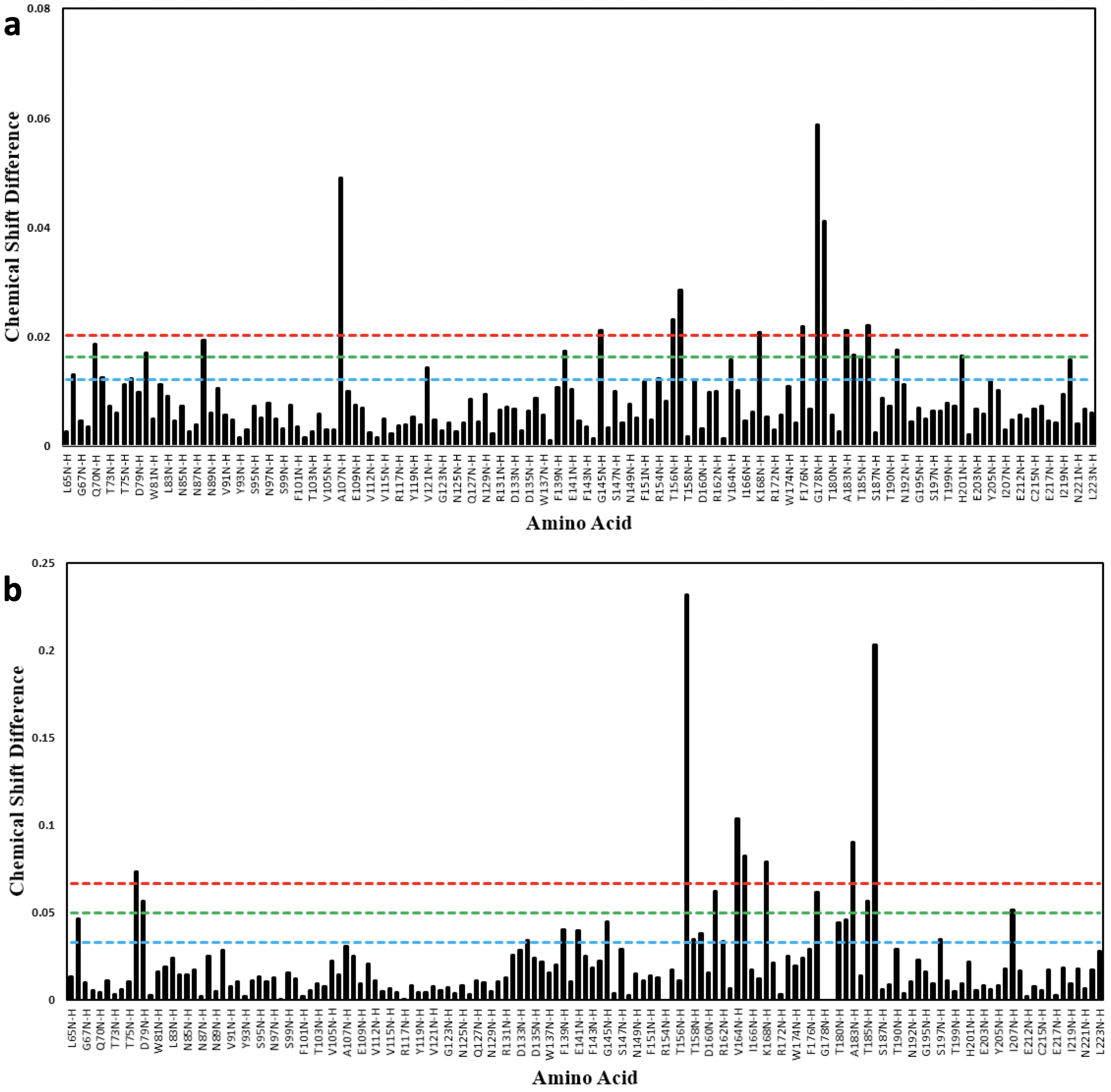

Supplement: S3 Fig — (a) Euclidean chemical shift changes of amino acids in P[8] VP8* upon addition of Leb tetra-saccharide. Blue line represents the standard deviation 1.5σ of Euclidean chemical shift change, and green and red lines represent 2σ and 2.5σ threshold values respectively. (b) Euclidean chemical shift changes of amino acids in P[8] VP8* upon addition of LNDFH I. Blue line represents the standard deviation σ of Euclidean chemical shift change, and green and red lines represent 1.5σ and 2σ threshold values, respectively. (TIF) [file ppat.1008386.s003.tif]

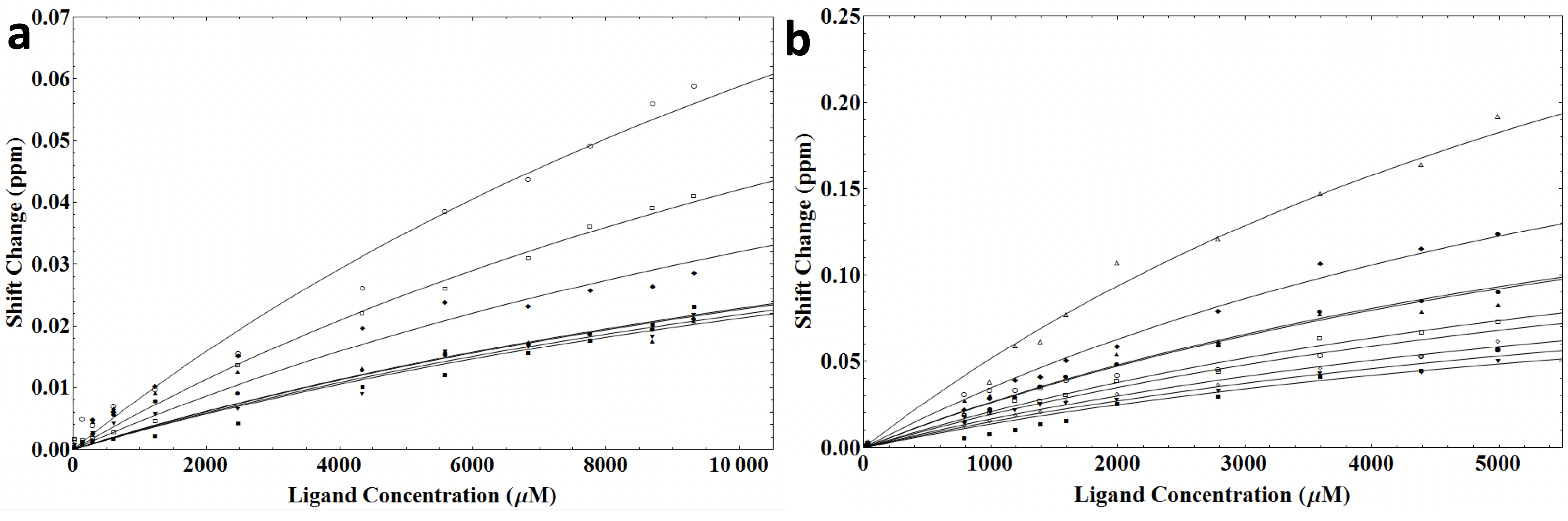

Supplement: S4 Fig — Global fitting for (a) P[8] and Leb tetra-saccharide, samples of 0.31 mM 15N-labeled P[8] VP8* was titrated with Leb tetra-saccharide at 0, 0.031, 0.15, 0.31, 0.62, 1.24, 2.49, 4.35, 5.60, 6.84, 7.78, 8.71, 9.33 mM and (b) P[8] and LNDFH I, Samples of 0.20 mM 15N-labeled P[8] VP8* was titrated with LNDFH I at 0, 0.020, 0.040, 0.80, 1.00, 1.20, 1.40, 1.60, 2.00, 2.80, 3.60, 4.40, 5.00 mM. (TIF) [file ppat.1008386.s004.tif]

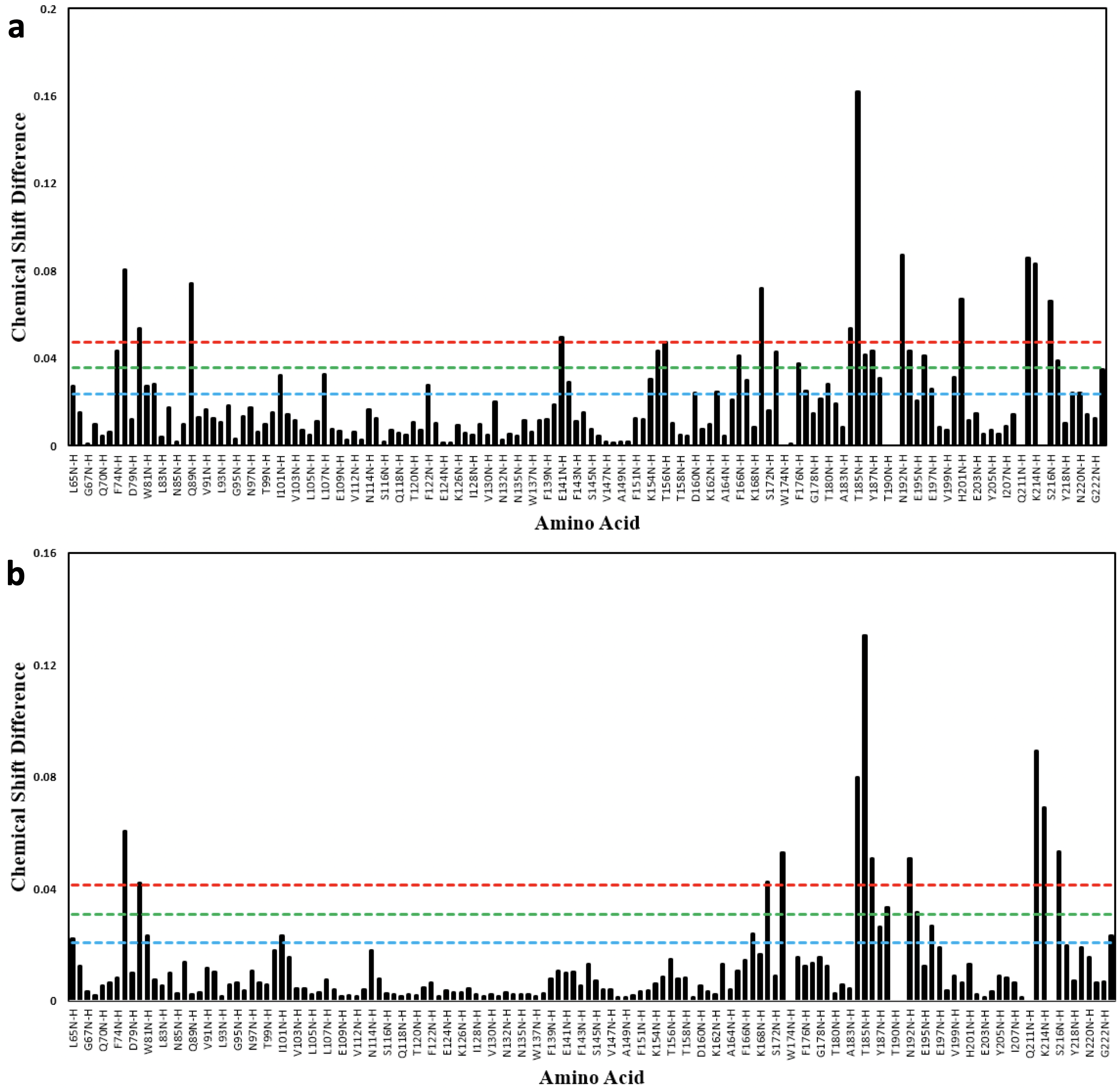

Supplement: S5 Fig — (a) Euclidean chemical shift changes of amino acids in P[6] VP8* upon addition of LNT. (b) Euclidean chemical shift changes of amino acids in P[6] VP8* upon addition of LNFP I. Blue line represents the standard deviation σ of Euclidean chemical shift change, and green and red lines represent 1.5σ and 2σ threshold values, respectively. (TIF) [file ppat.1008386.s005.tif]

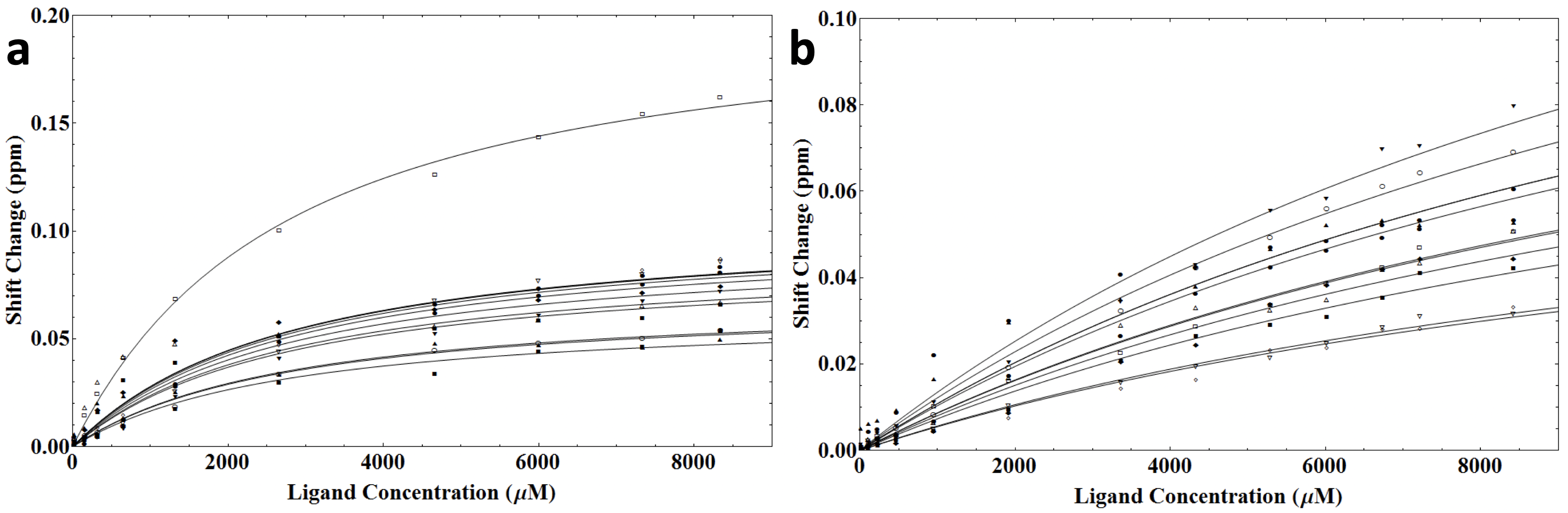

Supplement: S6 Fig — Global fitting to extract the dissociation constants from NMR titration for (a) P[6] with LNT. Samples of 0.33 mM 15N-labeled P[6] VP8* was titrated with LNT at 0, 0.033, 0.17, 0.33, 0.69, 1.34, 2.67, 4.68, 6.01, 7.35, 8.35 mM. and (b) P[6] with LNFP I. Samples of 0.24 mM 15N-labeled P[6] VP8* was titrated with LNFP I at 0, 0.024, 0.12, 0.24, 0.48, 0.96, 1.93, 3.37, 4.34, 5.30, 6.03, 6.75, 7.23, 8.44 mM. (TIF) [file ppat.1008386.s006.tif]

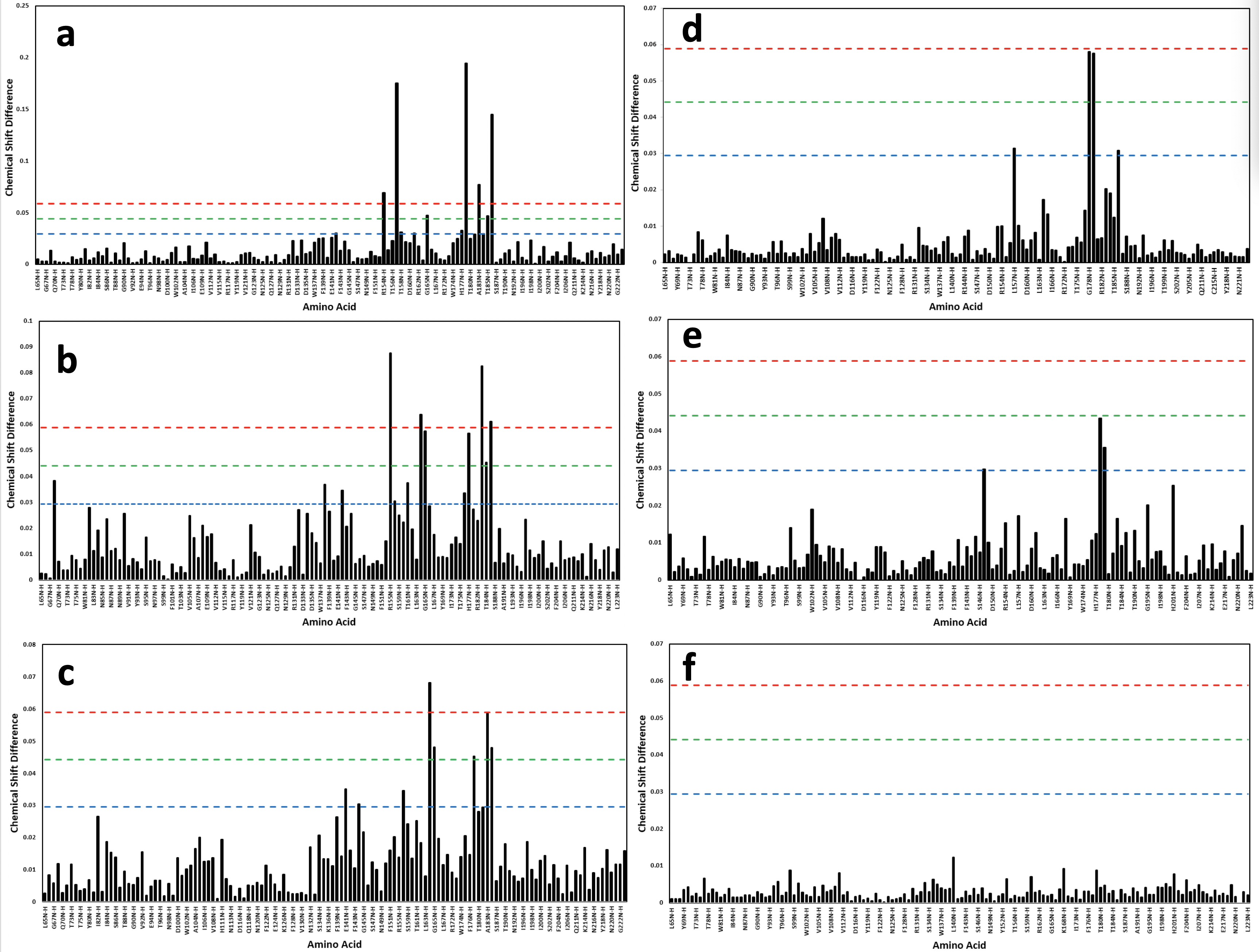

Supplement: S7 Fig — Euclidean chemical shift changes of amino acids in different P[8] VP8* mutants upon addition of LNDFH I for (a) K168A-P[8], (b) D79A-P[8], (c) T78A-P[8], (d) R154A-[8], (e) D186A-P[8], (f) H177A-P[8]. The threshold was obtained from the titration of the same ratio of LNDFH I into wt-P[8], with blue line represents the standard deviation σ of Euclidean chemical shift change, and green and red lines represent 1.5σ and 2σ threshold values, respectively. (TIF) [file ppat.1008386.s007.tif]

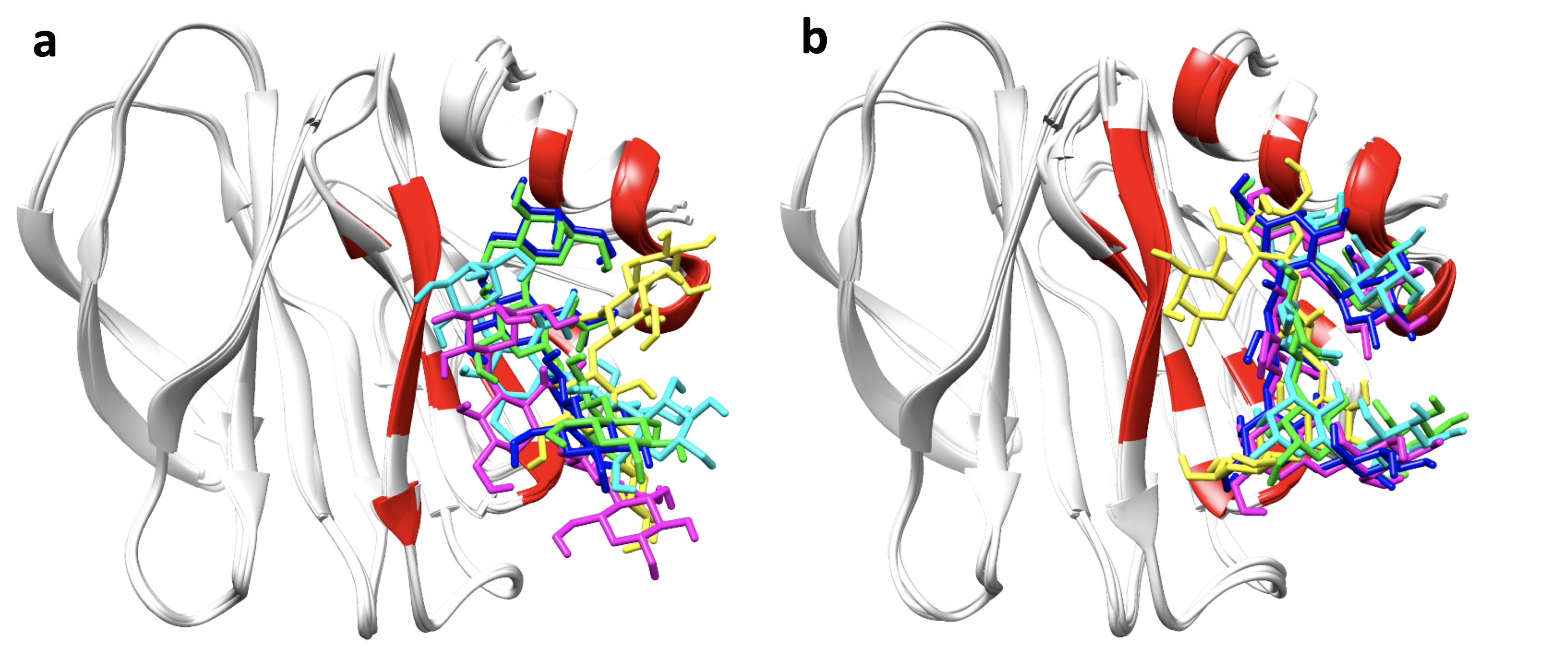

Supplement: S8 Fig — (a) Cartoon figures show the superposition of the top five best-scoring LNT bound P[6] VP8* structures from HADDOCK docking. (b) The superposition of the top five best-scoring LNFP I bound P[6] VP8* structures from the HADDOCK docking results. Red colors represent the binding interface. (TIF) [file ppat.1008386.s008.tif]

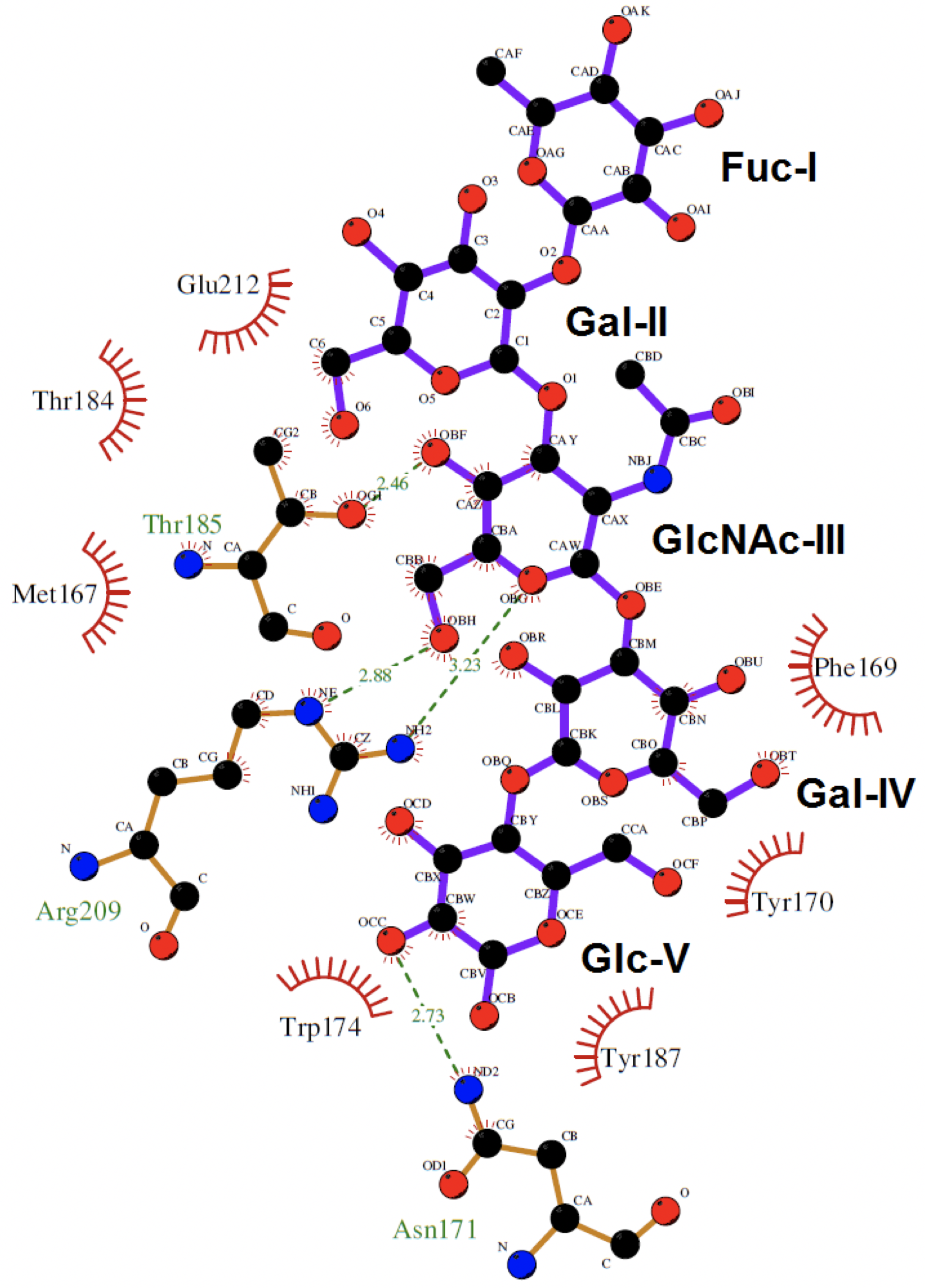

Supplement: S9 Fig — The diagram shows the specific interaction between P[6] VP8* and LNFP I. All the amino acid residues and saccharide moieties involved in the interactions are labeled. Hydrogen bond interactions are shown as green dashed lines between the respective donor and acceptor atoms along with the bond distance. The van der Walls contacts are indicated by an arc with spokes radiating towards the ligand atoms they contact. (TIF) [file ppat.1008386.s009.tif]

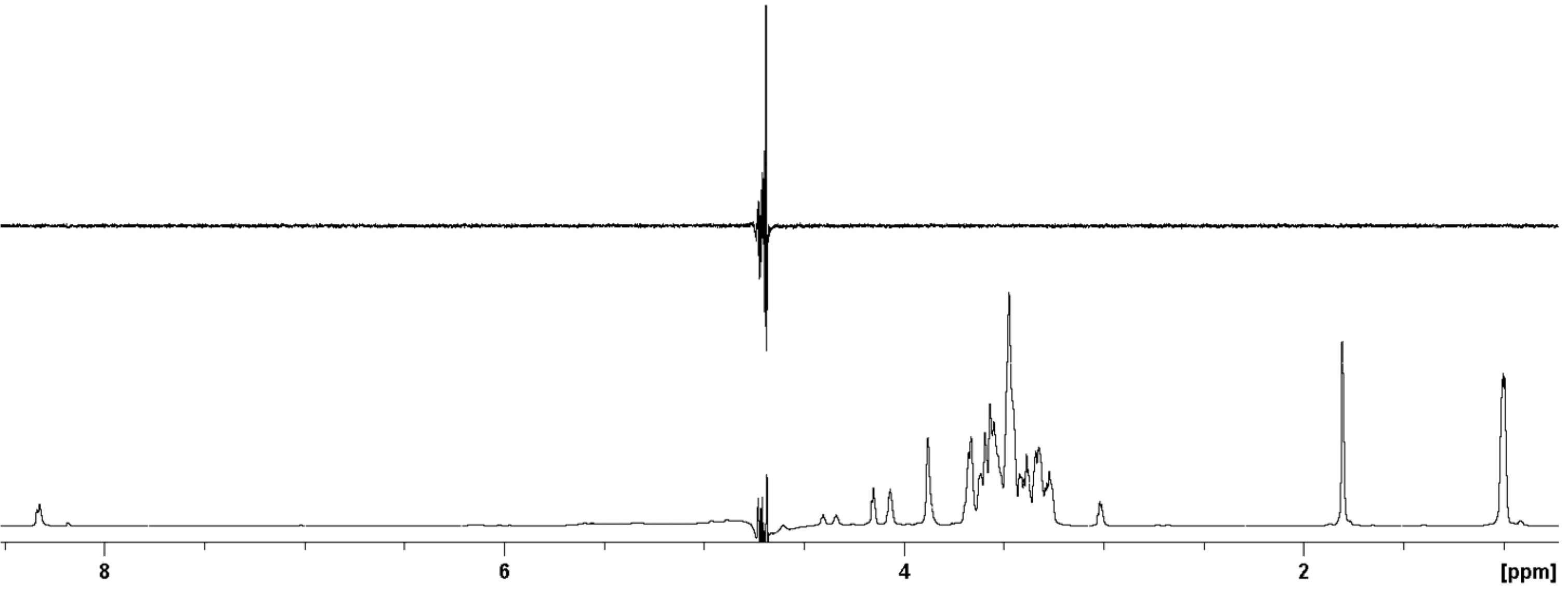

Supplement: S10 Fig — The bottom spectrum in (a) is the 1H NMR reference spectrum of glutathione S-transferase (40 μM) with LNDFH I (2.0 mM). The upper spectrum in (a) is the STD NMR spectrum of GST (40 μM) with LNDFHI (2.0 mM). The protein was saturated with a cascade of 40 Gaussian-shaped pulses at 6.8 ppm., and The off-resonance was set to 50 ppm. (TIF) [file ppat.1008386.s010.tif]

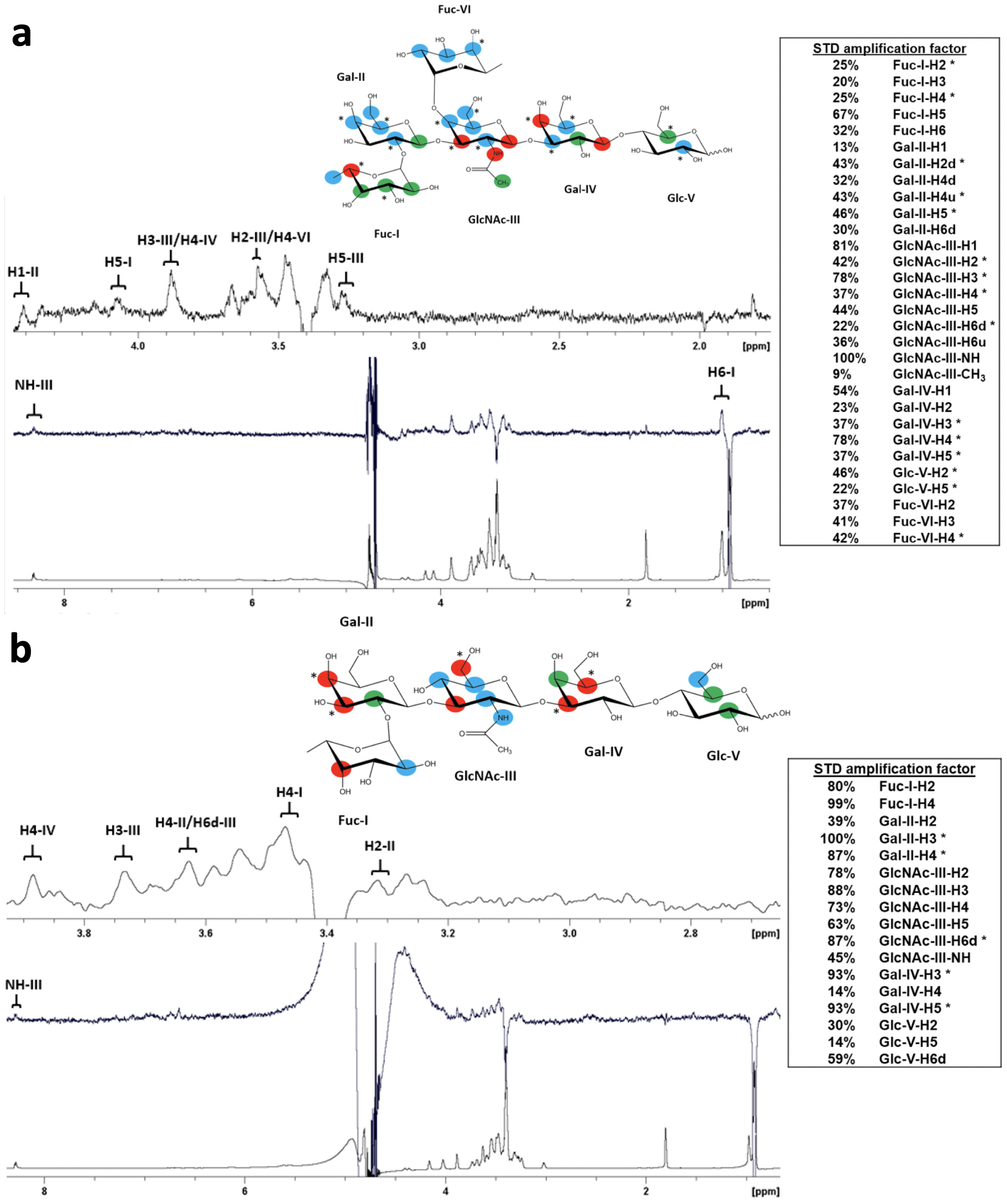

Supplement: S11 Fig — (a) NMR spectra pf LNDFH I in complex with P[4] VP8*. The bottom spectrum in (a) is the 1H NMR reference spectrum of P[4] VP8* (50 μM) with LNDFH I (2.5 mM).The middle spectrum in (a) is the STD NMR spectrum of P[4] VP8* (50 μM) with LNT (2.5 mM). The protein was saturated with a cascade of 40 Gaussian-shaped pulses at -0.20 ppm, and the off-resonance was set to 50 ppm. The upper spectrum in (a) is the expansion of the STD NMR from 4.0 to 2.6 ppm. (b) The epitope map of LNDFH I when bound to P[4] based on the STD effects: red, strong STD NMR effects (>50%); blue, medium STD NMR effects (30%-50%); green, weak STD NMR effects (<30%). (c) NMR spectra pf LNFP I in complex with P[4] VP8*. The bottom spectrum in (c) is the 1H NMR reference spectrum of P[4] VP8* (50 μM) with LNFP I (2.5 mM). The middle spectrum in (c) is the STD NMR spectrum of P[4] VP8* (50 μM) with LNFPI (2.5 mM). The protein was saturated with a cascade of 40 Gaussian-shaped pulses at -0.20 ppm, and the off-resonance was set to 50 ppm. The upper spectrum is the expansion of the STD NMR from 4.0 to 2.6 ppm. (d) The epitope map of LNFP I when bound to P[4] based on the STD effects: red, strong STD NMR effects (>85%); blue, medium STD NMR effects (40%-85%); green, weak STD NMR effects (<40%). “*” means the overlap of signals in the STD NMR spectrum. “d” indicates a downfield proton, “u” indicates an upfield proton. (TIF) [file ppat.1008386.s011.tif]

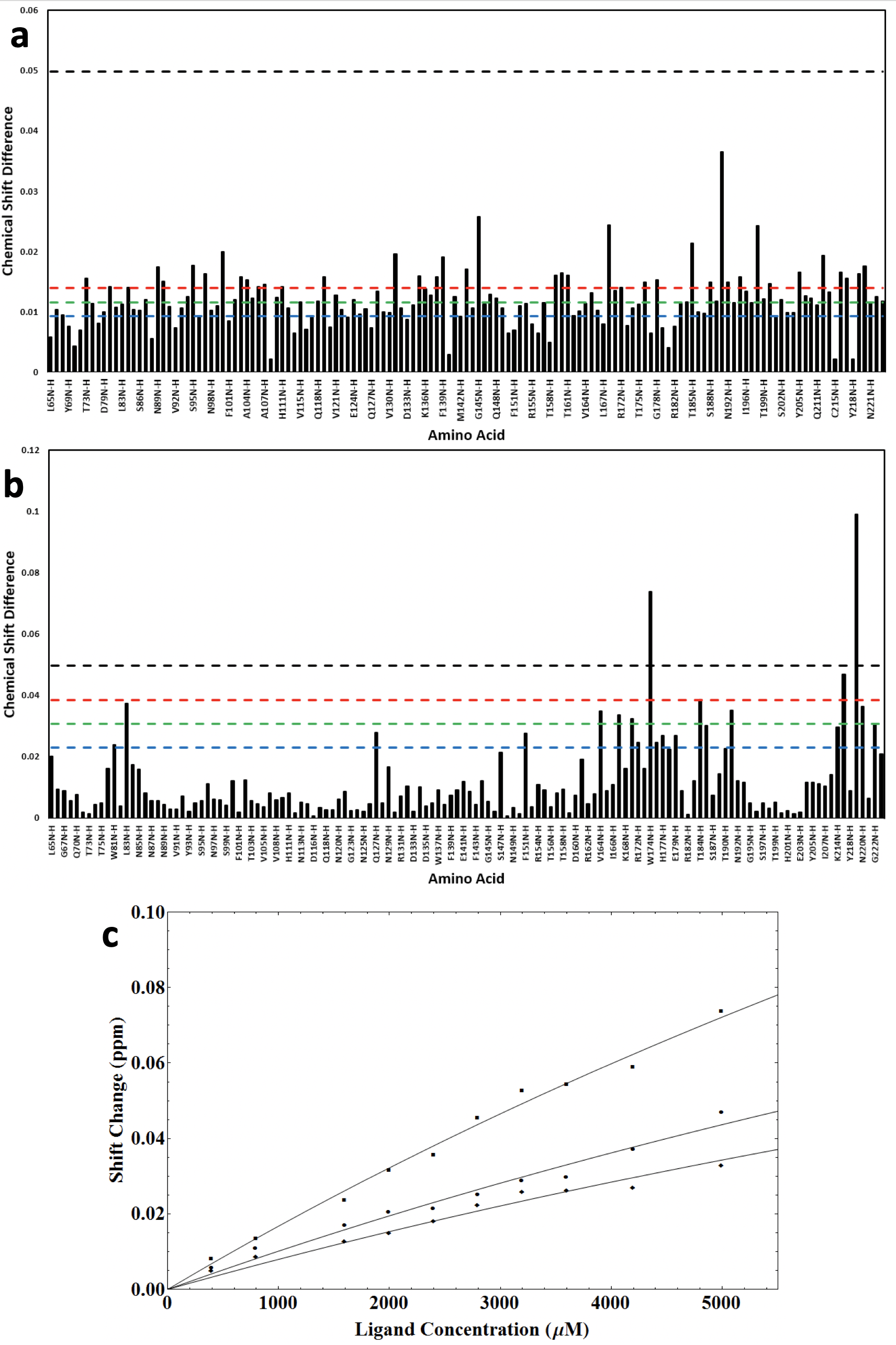

Supplement: S12 Fig — (a) Euclidean chemical shift changes of amino acids in P[8] VP8* upon addition of LNT. (b) Euclidean chemical shift changes of amino acids in P[8] VP8* upon addition of LNFP I. Blue line represents the standard deviation σ of Euclidean chemical shift change, and green and red lines represent 1.5σ and 2σ threshold values respectively. Black line represents the 1.5σ of Euclidean chemical shift change of P[8] upon addition of LNDFH I. (c) global fitting for P[8] and LNFP I, to get the dissociation constant. Samples of 0.20 mM 15N-labeled P[8] VP8* was titrated with LNFP I at 0, 0.020, 0.040, 0.80, 1.00, 1.20, 1.40, 1.60, 2.00, 2.80, 3.60, 4.40, 5.00 mM. (TIF) [file ppat.1008386.s012.tif]

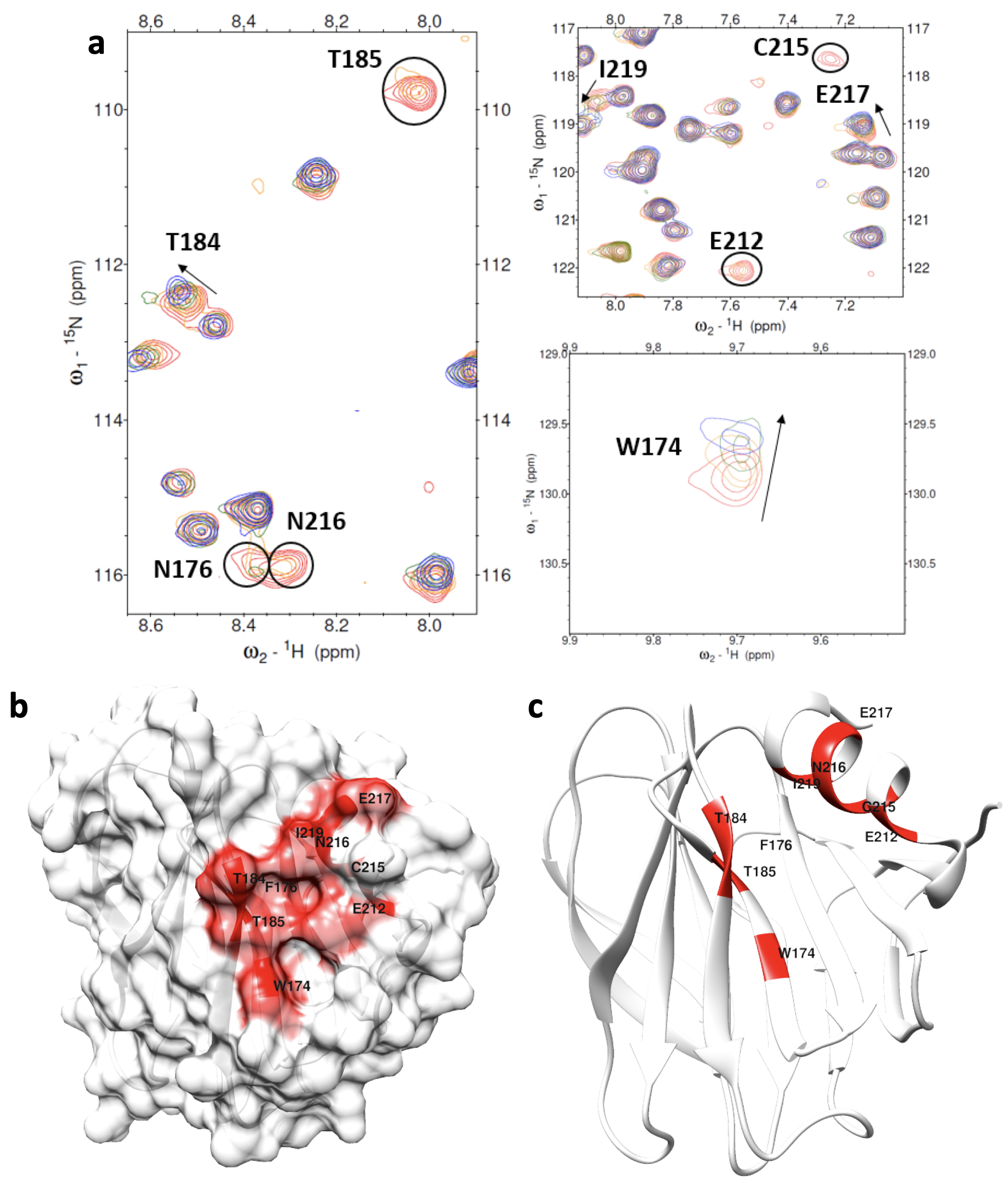

Supplement: S13 Fig — (a) Chemical shift changes in P[8] VP8* [PDB ID: 2DWR] upon addition of LNFP I. Titrations were followed by acquisition of two-dimensional 1H-15N HSQC spectra of mM 15N-labeled P[8] VP8*. The NMR data correspond to increasing ligand/protein ratios of 0:1 (red), 8:1 (orange), 18:1 (green), and 25:1 (blue). (b) Location of the large chemical shift changes on the P[8] VP8* surface [PDB ID: 2DWR] upon binding to LNFP I. Amino acids with chemical shift changes greater than 2σ or disappeared after titration are colored with red. (c) Ribbon Diagram shows the location of large chemical shift changes on the P[8] VP8* surface upon binding to LNFP I. The secondary structure is labeled. (TIF) [file ppat.1008386.s013.tif]
