# Supplementary material for: Molecular basis of P[II] major human rotavirus VP8* domain recognition of histo-blood group antigens
Source: PLoS Pathog. 2020 Mar 24;16(3):e1008386. doi: 10.1371/journal.ppat.1008386 (PMC7122821; doi:10.1371/journal.ppat.1008386)
Supplement: Attachment — Submitted filename: response-letter-1-28-20_final.docx [file ppat.1008386.s016.docx]

January 28, 2020

Editor

PLoS Pathogens

Dear Editor,

Please consider our revised manuscript entitled “**Molecular basis of P[II] major human rotavirus VP8* domain recognition of histo-blood group antigens**” for publication in *PLoS Pathogens*.

Below, we detail our responses to the reviewer's comments.

**Editor's instructions:**

**Response:** In the revised manuscript, we now cite and discuss the findings reported in recently published papers, including papers by Gozalbo-Rovira et al. PLoS Pathog 2019 and Sun X. et al, Virol Sin 2019. To reconcile the difference between the results reported here in our manuscript with the previous findings, we performed an additional HSQC titration of P[8] with LNFP I, and the result was found to be in agreement with the previous finding. The different binding affinities of different P[8] strains to specific HBGAs is discussed in the revised manuscript. The biological importance of the Lewis b-containing glycan fragments used in the NMR study presented in the manuscript was confirmed by infectivity experiments that demonstrated that addition of each glycan conjugated to either BSA or PAA inhibited P[8] replication, indicating that the Lewis b glycans were essential attachment factors for infection. In addition, we have also corrected minor issues in the manuscript, for example, fixing the text to reflect the fact that the number of VP4 genotypes now greater than 50.

Below, we respond to reviewers’ specific comments.

**Part II – Major Issues: Key Experiments Required for Acceptance**

**Reviewer #1:**

1. The paper by Gozalbo-Rovira published this year in PLoS Pathogens (<https://doi.org/10.1371/journal.ppat.1007865>) should be cited and critically discussed since it describes the carbohydrate-binding site of a P[8] VP8* through co-crystallization with the type 1 precursor disaccharide or the H type 1 trisaccharide. It involves both the alpha helix and beta strands, consistent with the conclusions of Hu et al, cited as reference 36. These two papers claim that the P[8] and P[6] binding sites are similar and that the fucose alpha1,4 linked to the GlcNAc residue of the type 1 precursor clashes in that binding site, hampering recognition of Lewis type glycans. This is consistent with the authors’ present data regarding P[6] VP8* as well as the above-mentioned epidemiological study of Nordgren et al and the saliva binding data reported by Barbé et al (Scientific Reports, 2018) also regarding P[6] VP8*. However, it is clearly at odds with the authors’ conclusions and with other previous studies that showed binding of P[8] VP8* to the difucosylated Lewis b as well as to saliva from Lewis b individuals but not to saliva from individuals lacking Lewis b (Huang et al J Virol 2012 ; Barbé et al Scientific Reports 2018). It appears that in absence of the alpha1,4-linked fucose residue, P[8] VP8* interact with type 1 oligosaccharides (based on lacto-N-biose) at the same site as P[6] VP8*, whilst the presence of that Lewis fucose, as shown here, shifts positioning of the oligosaccharide toward the two beta sheets mode. That should have a major impact on the virus attachment to natural glycans and the ensuing of infection and needs being properly discussed.

**Response:** We cited the publication by Sun et al J Virol. 2018;92(14) in our last submission of the manuscript and the publication by Gozalbo-Rovira in 2019 in PLoS Pathogens has been added. The crystal structures of P[8] VP8* in complex with the type 1 precursor and the H type 1 trisaccharide revealed that it recognized those two glycans through the interface involving both the beta strands and alpha helix, consistent with P[4], P[6], P[19] in P[II] genogroup in binding H type 1 glycans. We also titrated H type 1 antigen LNFP I to P[8] and found that P[8] recognized LNFP I using the same beta-alpha binding site. We included these new results and discussed our interpretation of the results which agrees with the reviewer’s suggestion that the study may“impact on the virus attachment to natural glycans and the ensuing of infection". Our interpretation is that P[8] interacts with type 1 HBGA precursor at the same site as P[4], P[6], and P[19], but it also interacts with the more matured Lewis b (LNDFH I) antigens in the beta beta site. This interpretation also provides an example of pathogen-host co-evolution of RVs under selection of the step-wisely synthesized HBGAs in humans and different animals leading to species-specific host ranges of individual P[II] genotypes to different human populations and some animal species determined by their genetic makeups of type 1 HBGAs, which is also discussed in the discussion section of the revised manuscript.

2. authors confirm here previously reported binding of P[8] VP8* to both Lewis b and H type 1. However, the same VP8* attach to saliva samples from Lewis b expressing individuals only and Lewis negative individuals appear largely spared by P[8] rotavirus. How can this be reconciled since an effect of the secretor status only should be visible if binding to H type 1 and Lewis b are equivalent? Could the STD effect observed with the Fuc-IV fucose residue (Fig. 2) be sufficient to increase affinity? A comparison of the dissociation constant for LNF I with that reported for LNDFH I is necessary to strengthen the manuscript by clarifying this issue.

**Response:** To clarify the reviewer’s concern, we performed HSQC titration of P[8] VP8* to LNT (Galβ1-3GlcNAcβ1-3Galβ1-4Glc) and LNFP I (Fucα1-2Galβ1-3GlcNAcβ1-3Galβ1-4Glc) and compared with the results to LNDFH I performed in our last submission of the manuscript. We found that P[8] VP8* had little binding signal to LNT but interacted with LNFP I by the βα mode with a significantly weaker affinity (23.49 mM ± 7.62 mM) than LNDFH I (6.29 Mm ± 0.76 mM ) by the ββ mode. Thus, the secretor fucose did played a role in the binding of LNFP I, while the Lewis fucose is critical for the binding of LNDFH I. Since only ~10% of the general population are Lewis type negative, and only ~8% of them are secretor type positive and can bind P[8] VP8*, which could be the reason being spared by the saliva binding assays.

**Reviewer #2:**

3. None

**Reviewer #3:**

4. By using STD-NMR, the authors showed that Leb and LNDFH1 bound to P[8] VP8* mainly use the type-1 precursor motif, while the Lewis and secretor fucose residues are also involved in the interaction. Infectivity experiments using Leb, LNDFH1, and a negative control glycan should be carried out to show the biological relevance of this study.

**Response:** From the STD-NMR experiments, we found that the type-1 precursor motif, and the Lewis and secretor fucose residues are involved in the interaction. We conducted the infectivity experiments using LNFP I, LNDFH I, and the negative control glycan (Le^y^ antigen). The biological importance of the Lewis b-containing glycan fragments used in the NMR study presented in the manuscript was confirmed by infectivity experiments that demonstrated that addition of each glycan conjugated to either BSA or PAA inhibited P[8] replication, indicating that the Lewis b glycans were essential attachment factors for infection.

**Part III – Minor Issues: Editorial and Data Presentation Modifications**

**Reviewer #1:**

5. The authors compared X-ray crystallography and NMR analyses for the P[6] VP8*-glycan interaction. Why not perform an X-ray crystallography analysis of the difucosylated oligosaccharides with a P[8] VP8*? If that failed, it would be worth mentioning.

**Response:** Yes, we have tried to co-crystallize P[8]-LNDFH I and P[8]-Lewis b tetra-saccharide without success to this point, possibly due to a combination of weak binding and large binding interface of the VP8* domain with the two long glycans making it difficult to co-crystallization. This effort is ongoing in our laboratory.

6. In the abstract with several additional occurrences in the manuscript, the authors state that HBGAs have been identified as receptors for human RV strains. It would be wiser to speak about attachment factors rather than receptors since these glycans do not seem to be required for in vitro infection.

**Response:** We have changed from “the HBGAs as receptors” to “attachment factors” in the manuscript.

7. Legend to Fig 2 indicates P[6] instead of P[8], please correct.

**Response:** The Legend has been corrected to P[8].

8. On page 15, first paragraph, Fig 9c should be Fig 10.

**Response:** We have corrected it to Fig 10c.

9. On page 15, the last sentences of the results section require language edition.

**Response:** We have rearranged the sentence as follows. “Because amino acids in the ββ binding site of the BM13851 strain of P[8] are same as those residues in the Wa strain and other strains of P[8], we predicted that other strains of P[8] can recognize the Lewis-containing LNDFH I in the same ββ site as the BM13851 strain of P[8]. ”

10. On page 17, the difference in dissociation constants between Lewis b tetrasaccharide and LNDFH I is used to argue that P[8] and P[4] strains have adapted to recognize more mature type 1 HBGAs. However, the affinity difference is more likely due to the contribution of the lactose unit in LNDFH I. The Leb tetrasaccharide does not correspond to a naturally occurring molecule. Therefore an affinity difference between the hexasaccharide and tetrasaccharide forms of Leb cannot be ascribed to any particular adaptation. Nonetheless, I agree that an adaptation likely took place to accommodate fucosylated glycans by shifting from the βα to the ββ binding domains.

**Response:** We agree with reviewer’s comments. The adaptation is not due to the different dissociation constants between tetrasaccharide and hexasaccharide forms of Le^b^, but likely because P[8] can accommodate difucosalyted glycans by shifting from the βα to the ββ binding. Based on our new titration data, we have changed the discussion: “The binding affinity of P[8] to the Lewis positive hexa-saccharide LNDFH I is much tighter than to the Lewis negative penta-saccharide LNFP I, and it accommodate the difucosalyted glycans by shifting from the βα to the ββ binding, supporting the view that P[8] and P[4] are more distantly evolved from their P[I] ancestor and have adapted to recognize more mature type 1 HBGAs”.

11. References numbering should be checked as there are errors. For example, reference 45 cited in the text as Jiang et al is #46 in the reference list.

**Response:** We have checked the references, and updated the reference from Jiang et al.

12. Reference 27 (Ayouni et al) is cited as showing that nonsecretors individuals may be resistant to P[8] and P[4] infections. Unfortunately, that study reported a lack of association between the secretor phenotype and rotavirus infection. It should be correctly cited or replaced by another reference corresponding to a study reporting the correct association between the secretor status and P[8] infection.

**Response:** We have updated the discussion about the results from Ayouni et al, and included the discussion of other studies regarding the association between the secretor status and P[8] infection.

13. From the colors on Fig 7, only 4 modeled Leb or LNDFH I can be distinguished whilst 5 are mentioned in the text and legend to the figure? Is it simply a difficulty to visualized 5 superimposed structures and can this be improved?

**Response:** To improve the visualization, we changed the ligands from the “ball & stick” form to “stick” form, and used five colors, yellow, green, blue, cyan, magenta, to represent the ligands.

14. It would be useful to expand the Lewis b binding area shown on Fig 7c in order to better visualize the positioning of the two fucose residues.

**Response:** To better visualize the position of the two fucose residues, the best HADDOCK soring P[8] VP8* models were zoomed in to highlight the binding pockets. Each carbohydrate moiety of the ligand was colored differently.

15. Adding the meaning of the red and green colors assigned to individual resonance assignments on Fig S1 would help the reader.

**Response:** We have added the meaning of the red and green colors to the HSQC spectra. Red colors indicate positive signals, green colors indicate negative signals.

**Reviewer #2:**

16. The bmrb accession code given on p.9, l. 2 is certainly not correct

**Response:** The BMRB accession code in the manuscript is the BMRB entry ID number, we have corrected the ID number in the manuscript. The P[8] entry ID is 27592, and the P[6] entry ID is 27591. We have corrected the entry ID for P[8] in the manuscript.

17. Some of the dissociation constants reported are in the two-digit millimolar range. Can the authors comment in some more detail - maybe in the supplementary part - up to which concentration they have done the CSP titrations. Some of the titration curves look rather "linear".

**Response:** To improve the fit, the data were globally fitted to get the binding affinity through simultaneously fitting amino acids in the interface that showed large chemical shifts. Considering the availability of the ligands, the solubility of the ligands, and the solubility of the complex in the buffer, we usually ended the titration up to 1:25 or 1:35 ratio that corresponded to 5mM to 10 mM of ligands, and we included the concentrations of ligands in the supplementary materials. Due to the nature of weak binding, some curves look “linear”, and it also gives us relative quantified information with some dissociation constants reported in the two-digit millimolar range such that we can compare relative affinities.

**Reviewer #3:**

18. The authors referred to the glycan-binding sites as “beta-alpha binding domain” and “beta-beta binding domain”. However, protein domains, such as VP8* domain, form compact 3D structures that are independently stable and folded. Have the authors expressed the “beta-alpha” sheets or “beta-alpha” independently? If not, it is more accurate to refer to them as “sites” instead of “domains”.

**Response:** We changed the “beta-alpha binding domain” and “beta-beta binding domain” to “beta-alpha binding site” and “beta-beta binding site”.

19. The authors cited "Fig1a" or "Fig1c" in the text. However, there are no "a","b"..lables in Figure 1.

**Response:** We added the labels in figure 1.

20. The crystallographic statistics table (Page 12-14) is out of place and does not have a table legend.

**Response:** We have rearranged the crystallographic statistics table and added the table legend.

We hope that our revised manuscript is now considered suitable for publication in *PLoS Pathogens*. We look forward to continued correspondence regarding publication of our manuscript.

Sincerely,

Michael A. Kennedy, PhD

Eminent Scholar and Professor

Department of Chemistry and Biochemistry

106 Hughes Laboratory

Miami University Oxford, OH, 45056
